# Incidence of Post-ablation Syndrome Following Image-Guided Percutaneous Cryoablation of Renal Cell Carcinoma: A Prospective Study

**DOI:** 10.1007/s00270-017-1811-1

**Published:** 2017-11-28

**Authors:** Jim Zhong, Janette Bambrook, Balbir Bhambra, Jonathan Smith, Jon Cartledge, Christy Ralph, Naveen Vasudev, Simon Whiteley, Tze Wah

**Affiliations:** 1 Diagnostic and Interventional Radiology Department, Institute of Oncology, St. James’s University Hospital, Leeds Teaching Hospitals NHS Trust, Leeds, LS9 7TF UK; 2 Department of Urology, St. James’s University Hospital, Leeds Teaching Hospitals NHS Trust, Leeds, LS9 7TF UK; 3 Department of Medical Oncology, Institute of Oncology, St. James’s University Hospital, Leeds Teaching Hospitals NHS Trust, Leeds, LS9 7TF UK; 4 Department of Anaesthesia, Institute of Oncology, St. James’s University Hospital, Leeds Teaching Hospitals NHS Trust, Leeds, LS9 7TF UK

**Keywords:** Post-ablation syndrome, Cryoablation, Radiofrequency ablation, Renal cell cancer

## Abstract

**Purpose:**

To prospectively evaluate the incidence of post-ablation syndrome (fever and flu-like symptoms) and impact on the quality of life in the first 10 days following percutaneous image-guided cryoablation for renal cell carcinoma (RCC).

**Materials and Methods:**

A prospective study of all cryoablation procedures with biopsy proven RCC was conducted with institutional review board approval between 08/2012 and 04/2016. Sixty-four patients (43 males and 21 females) underwent cryoablation. Mean age was 68 (range 24–86). A telephone questionnaire survey was conducted on days 1, 3, 5, 7 and 10 following cryoablation, and complications were recorded. Data collected included temperature, degree of flu-like symptoms, severity of pain, percentage of pain relief with analgesics, interference with general activity and with work (graded on a 0–10 Numeric Intensity Scale).

**Results:**

Following cryoablation, six patients (9%) out of 64 developed post-ablation syndrome. Thirty-three patients (52%) developed flu-like symptoms only, which completely resolved by day 10 in 25 patients (39%). One patient had pyrexia only, which was self- limiting by day 10. Twenty-four patients (38%) were asymptomatic. Pain (mean score = 2.1) and interference on general activities (mean score = 1.8) and work (mean score = 2) following cryoablation peaked on day 3 and improved subsequently. Forty-six patients (72%) had 90–100% pain relief by day 10. No major complications were observed.

**Conclusion:**

The full spectrum of post-ablation syndrome following cryoablation occurs in approximately 9% of patients; however, 61% of patients experience flu-like symptoms in the first 10 days, which are self-limiting.

## Introduction

Over the last decade, percutaneous image-guided thermal ablation has emerged as a useful minimally invasive nephron-sparing therapeutic intervention to treat small cancers in a variety of organs, in particular the liver and kidney, where ablative techniques are now established [1–4].

The two principal methods of thermal ablation are using heat-based energy, e.g., radiofrequency ablation (RFA) or microwave ablation (MWA) and cryoablation, which is an ice-based energy to induce tissue necrosis and cancer cell death [1, 5]. Image-guided [6] cryoablation utilises rapid cooling of cryoprobes—small diameter (13–17 gauge) straight metallic shafts, within targeted lesions to cause cell necrosis by direct cellular injury and indirectly through changes in the cellular microenvironment [7]. Cryoablation has become favourable amongst clinicians, particularly for the treatment of patients with impaired renal function or with a solitary kidney [1, 8, 9].

For liver and kidney tumours, cryoablation has been shown to be safe and efficacious through data generated from cohort studies [10–14]. An important advantage of cryoablation over RFA is the excellent visualisation of the iceball on intraprocedural imaging such as computed tomography (CT) or magnetic resonance imaging (MRI). The size of the iceball correlates to tissue injury therefore cryoablation offers accurate monitoring of the extent of tissue damage and treatment margins to ensure adequate tumour coverage [12]. Recent data suggest that in the intermediate term, outcomes might be comparable to partial nephrectomy, which is still seen as the gold standard for small renal cancers (< 4 cm) [15].

Initial clinical experience of the technique shows, however, that some patients develop flu-like symptoms similar to the post-ablation syndrome described following RFA [16, 17] and post-embolisation syndrome seen in patients who have undergone pre-operative embolisation of liver or renal tumours [18–22].

This consists of low-grade fever, malaise, myalgia and nausea or vomiting, which occurs typically in the first 24–48 h after the procedure. The mechanism for this reaction has been speculated to stem from the production of cytokines as part of an inflammatory response to the necrotic tissues [6, 23, 24].

To our knowledge, there has not been any published data evaluating post-cryoablation syndrome in a large cohort of patients following cryoablation of renal cell cancer (RCC).

The primary aim of this study was to evaluate prospectively the incidence of post-cryoablation syndrome and to determine its impact on the quality of life in the first 10 days after cryoablation.

## Methods

### Study Design

This prospective study of all image-guided renal cryoablations in a single specialist oncology centre in the United Kingdom was conducted with institutional review board approval between August 2012 and April 2016. No ethics approval was required as the study was not classified as research under the United Kingdom National Health Service Health Research Authority. All patients were followed up with a standardised questionnaire to assess clinical response.

Only patients treated with cryoablation for biopsy proved RCC were included in the study cohort. Lesions that were not biopsied or benign histological findings were excluded from the study. Other exclusion criteria included previous locoregional therapy to the kidney. All procedures performed accordance with the ethical standards of the institutional and/or national research committee and with the 1964 Helsinki Declaration and its later amendments or comparable ethical standards. Informed consent was obtained for this survey in all cases by the interventional oncology programme coordinator and nurse.

### Cryoablation Pathway

All patients were recommended for cryoablation following multidisciplinary team (MDT) assessment and consensus agreement. The procedure was performed using a dedicated computed tomography (CT)-equipped interventional suite under general anaesthesia by a consultant interventional radiologist (TMW or JTS). Contrast-enhanced CT guidance allowed perioperative visualisation of the tumour and its margin through sequential scanning. All patients were scanned on a 64 slice MDCT scanner (Somatom Sensation 64, Siemens, Erlangen, Germany). For all scans, the tube kilovoltage (kVp) was fixed at 120 kV and was not adjusted according to patient size. Prior to insertion of the cryoprobes, three helical acquisitions were performed. The first was a non-contrast-enhanced scan, followed by arterial and portal venous phase acquisitions which were performed at 30 and 65 s, respectively, after injection of 100 mls of Niopam 300 intra-venous contrast agent (Bracco, Milan, Italy). This was to confirm the tumour volume based on the pre-procedural planning CT and determine the number of cryoprobes required for treatment. Adjunctive protective measures, including hydrodissection, pneumo-dissection or retrograde pyeloperfusion, were taken to prevent injury to surrounding structures. The technique for these has been described in a previous paper by Wah et al. [25].

A combination of 17G Ice Sphere and Ice Seed cryoprobes (Galil Medical, Arden Hills, MN, USA) were inserted percutaneously into the tumour under CT guidance, typically at the periphery, simulating a clock-face approach to sculpt the required ice ball to ensure an adequate zone of ablation and effective coverage of the tumour during treatment. Two complete cycles of freezing and thawing were performed (typically a 10 min freeze followed by 6 min thaw, then a 10 min freeze and 6 min thaw). One or several needle biopsies were obtained for histological confirmation immediate prior to cryoablation treatment.

After cryoablation, all patients were monitored in the post-general anaesthesia (GA) recovery area and vital observations such as blood pressure, heart rate, oxygen saturations and respiratory rate were monitored. Patients were discharged home the next day if they were haemodynamically stable and had recovered from GA to the point of tolerating oral intake and were passing adequate volumes of urine with stable renal function. Simple analgesia, such as orally taken paracetamol and codeine, was provided if required in the post-operative period.

Post-cryoablation complications were classified based on the Society of Interventional Radiology Classification System into minor and major complications [26]. Minor complications were defined as those of no clinical consequence or requiring none or minimal conservative therapy such as simple analgesia. Major complications included those, which affected clinical consequence requiring further therapy, hospitalisation, increase in level of care, permanent adverse sequelae or death.

### Clinical Response Survey After Cryoablation

A survey was conducted with a standardised questionnaire on days 1, 3, 5, 7 and 10 following their cryoablation procedure. Please see appendix section for the questionnaire. All inpatients and outpatients were surveyed with the same questionnaire. This survey was conducted by one of two interventional nurse/coordinators and carried out by telephone if the patient had been discharged or in person if the patient was still in hospital.

All patients were asked to monitor their temperatures in the morning when they first woke up and as needed, for the initial 10 days following their cryoablation. The patients were instructed to use a forehead thermometer for temperature measurements, which was given to them at discharge.

Clinical symptoms including temperature, flu-like symptoms, severity of pain, percentage pain relief with oral analgesics, type of oral analgesic, and any interference with general activity or work were recorded. All symptoms and interferences with lifestyle were graded 0–10 with a numeric intensity scale. Fever was defined as temperature > 37 °C.

### Post-ablation Syndrome

A full complete spectrum of post-ablation syndrome is defined as a combination of fever and flu-like symptoms, which includes myalgia, malaise and pain at the site of ablation. This is similar to the recognised post-embolisation syndrome and post-ablation syndrome following RFA, which has been defined in the literature [9, 16, 17, 19, 21].

### Statistical Analysis

Statistical analysis was performed with software (Minitab Express, State College, Pennsylvania, USA). Patients with post-ablation syndrome, the presence of either fever or flu-like symptoms or both were compared using a two-sample *t* test to assess whether the presence of fever influenced the severity/grade of the flu-like symptoms.

Simple linear regression analysis was used to see if there was any correlation between cryoablation time and severity of flu-like symptoms and pain. Analysis of variance was used to compare the number of cryoprobes with the severity of flu-like symptoms and post-procedure pain experienced to see if there was any correlation.

## Results

A total of 64 patients underwent cryoablation for biopsy proven RCC and met the inclusion criteria. Of these, 43 patients were male and 21 were female, with a mean age of 68 (range 24–86). One patient had two renal tumours cryoablated; therefore, a total of 65 renal lesions histologically confirmed as renal cell carcinoma (RCC) were ablated. The pathology of the specimens was clear cell RCC (*n* = 50); papillary type RCC (*n* = 8) and chromophobe type RCC (*n* = 7). The average size of the ablated renal lesion was 3.0 cm (range 0.9–6.8 cm). Mean CRYO time was 34.5 min (range 31–51 min), and mean number of cryoprobes used was 6 (range 2–12).

Three patients had adjunctive protective measures during cryoablation: Two patients had hydro-pneumo-dissection and one patient had hydrodissection.

No patient experienced major complications following cryoablation. Pain, fever and flu-like symptoms were the most common minor complications and will be discussed in the following sections.

### Post-ablation Syndrome

**Fig. 1:**
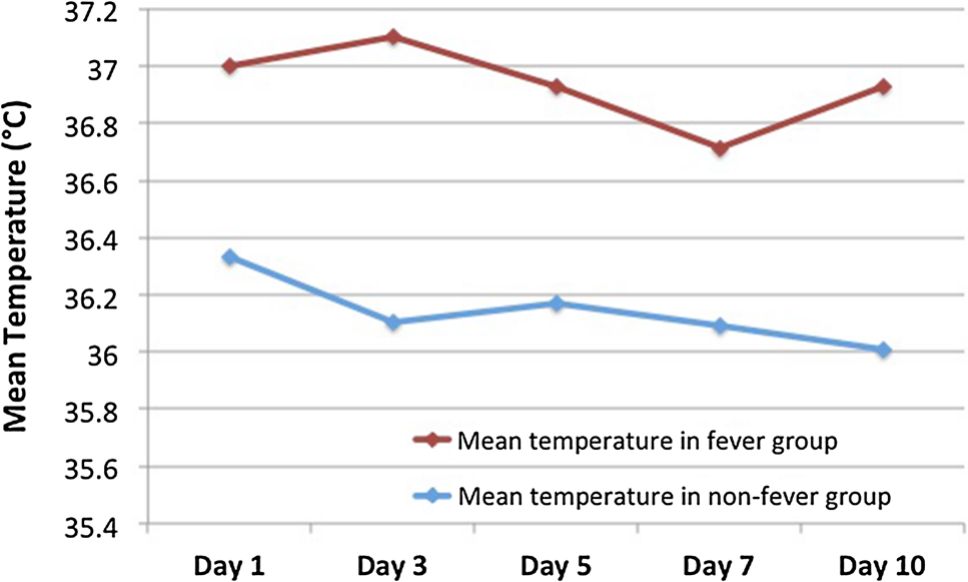
Graph showing mean temperature (in degrees Celsius) in patients after CRYO for renal lesion(s). To convert to Fahrenheit: [°F] = [°C] × 9/5 + 32

Seven patients (11%) in total developed a fever (temperature > 37 °C) in the first 10 days following cryoablation. The mean temperature in the first 10 days following cryoablation was 36.3 °C (range 35–38.5). The mean temperature of all patients who did not have fever compared with those who had fever is shown in Fig. 1.

The mean temperature in the group with fever peaked on day 3. A total of 39 patients (61%) experienced flu-like symptoms following cryoablation, with the peak in severity of symptoms on day 3. The mean score for flu-like symptoms (out of 10) in the first 10 days following cryoablation was 1.6 (range 0–8).

Six patients (9%) out of 64 developed complete post-ablation syndrome and experienced both fever and flu-like symptoms. These flu-like symptoms peaked on day 3 and resolved spontaneously in 5 out of the 6 cases. In the case where the flu-like symptoms did not resolve, the patient had remained an inpatient following cryoablation and developed their first pyrexial episode (temperature of 39 °C) on day 10, which was attributed to a chest infection and right foot cellulitis, which subsequently resolved with antibiotics.

Thirty-three patients (52%) developed flu-like symptoms only, without fever during the first 10 days following cryoablation. These flu-like symptoms had completely resolved by day 10 in 25 of the 33 patients. Of the eight patients who had residual flu-like symptoms on day 10, six rated the severity as only 1 or 2.

One patient developed fever without any flu-like symptoms. This resolved without any treatment by day 10.

Twenty-four patients (37.5%) did not have any flu-like symptoms or fever following cryoablation.

**Fig. 2:**
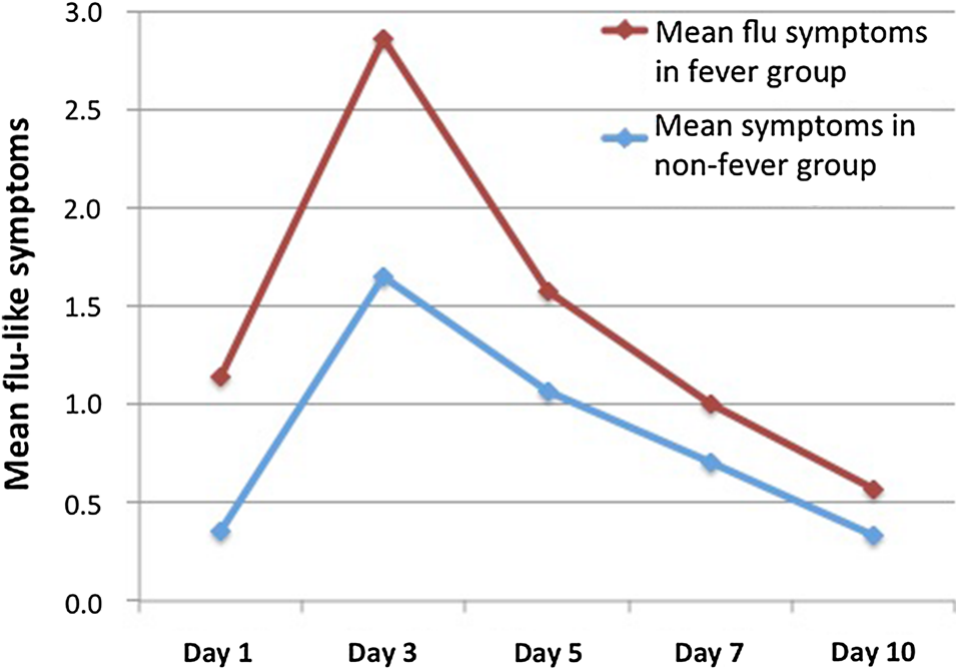
Graph showing the mean flu-like symptoms (0–10 numeric grading scale) in the patients who experienced fever compared to the mean flu-like symptoms in the patients who did not have fever

The mean scores for flu-like symptoms in the first 10 days following cryoablation in patients who had complete spectrum of post-ablation syndrome (*n* = 6) (fever and flu-like symptoms) compared with the flu-like symptoms in patients who did not have fever (*n* = 33) shows that the patients who had fever also experienced more severe flu-like symptoms as demonstrated by a higher mean score (Fig. 2), most marked on day 1 and 2. The severity of symptoms peaked in both groups on day 3. This, however, did not reach statistical significance following the *t* test (*P* > 0.05).

### Pain and Lifestyle Interference

The mean pain severity score following cryoablation was 2.1 (range 0–8) over the 10 days. Eight patients (13%) had no pain in the 10 days following cryoablation and 56 patients (87%) experienced some pain during the first 10 days, with the peak at day 3 where patients had a mean score of 2.8 out of 10. Thirty-eight patients (59%) did not require any analgesia for pain relief. Forty-six patients (72%) had 90–100% relief of their pain by day 10 with or without simple analgesics. Seventeen patients required only simple orally taken paracetamol for pain relief, and nine patients required orally taken ibuprofen or codeine in addition to paracetamol. The overall percentage of pain relief by day 10 with or without analgesics ranged from 0 to 100% (Mean = 8%).

**Fig. 3:**
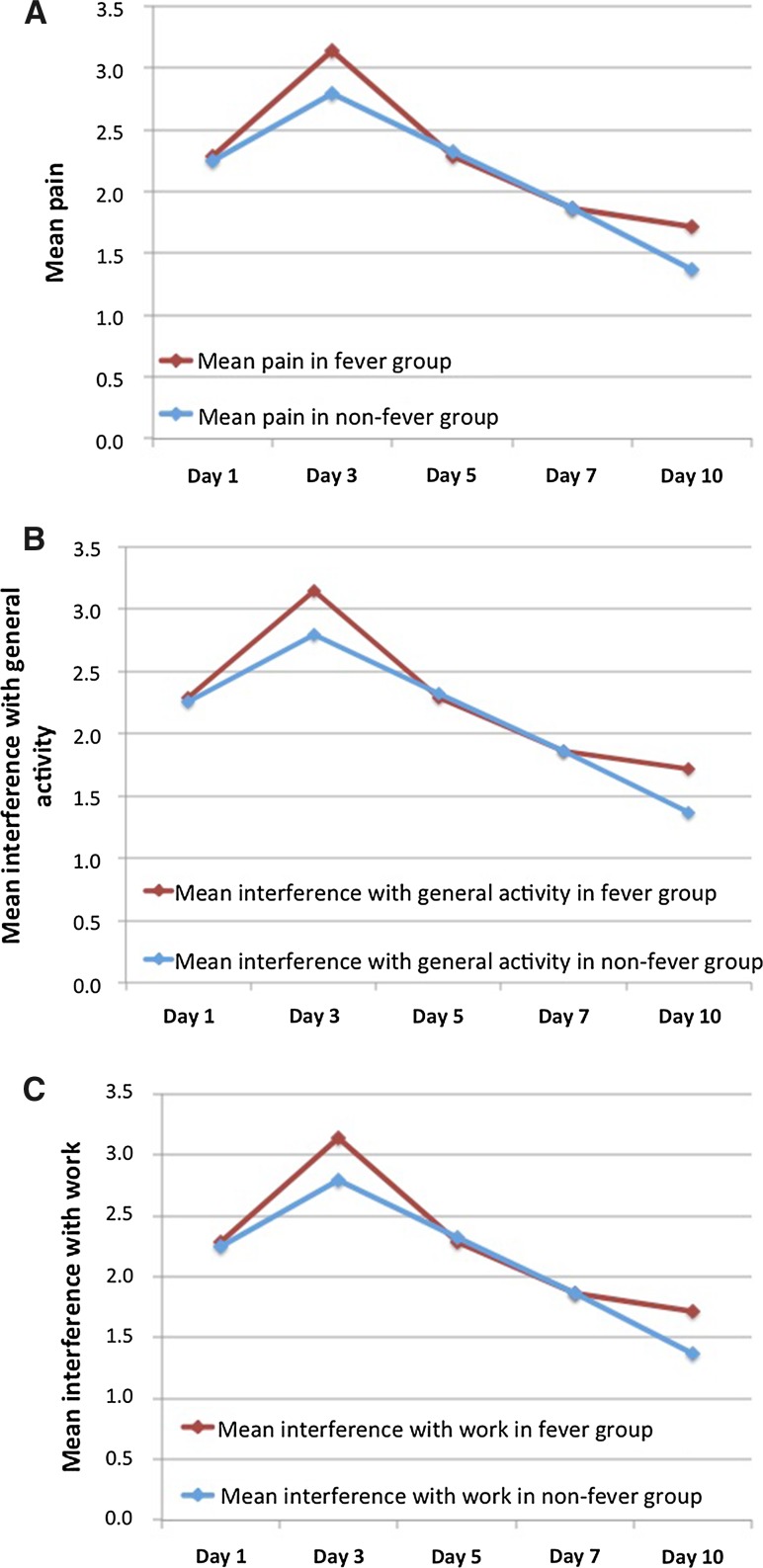
Graph showing: **A** Mean pain in the patients without fever and patients with fever (temperature > 37 °C). **B** Mean interference with general activity in the patients without fever and patients with fever (temperature > 37 °C). **C** Mean interference with work in the patients without fever and patients with fever (temperature > 37 °C)

Figure 3A shows the trend in mean pain experienced by patients without fever (mean score at day 3 was 2.8) and patients with fever (mean score at day 3 was 3.1) (temperature > 37 °C).

Between day 1 and day 10, the mean interference on general activities was 1.8 (range 0–10) and mean interference on work was 2.0 (range 0–10).

The interference on general activities and work activities following cryoablation peaked on day 3 for both categories. The mean score for interference on general activities at day 3 was 3.4, and the mean score for interference on work activities at day 3 was 3.2. Figure 3B, C shows the mean interference on general activities and work activities in the patients without fever and patients with fever (temperature > 37 °C). For the patients who had fever, there was a higher mean interference with general activities and work compared to the group of patients without fever; however, the trend was the same, with improvement from day 3 onwards.

No statistical correlation was found between the total cryoablation time or number of cryoprobes used with the severity of the flu-like symptoms or pain experienced (*P* > 0.05).

## Discussion

The primary thermal ablation techniques used for renal tumours are RFA and cryoablation. At our institution, all small renal tumours are treated with cryoablation. The clear advantages of cryoablation over RFA are direct visualisation of the ice ball under CT, allowing for precise monitoring of the ablation zone [27]. Randomised control trial data comparing RFA and cryoablation found in the cryoablation group there was a significantly lower local tumour progression rate, which could be due to better visualisation of the treatment margins to optimise oncological control [13]. CT guidance is preferred over ultrasound to allow for the exact anatomical relationship of the cryoprobes with the tumour and surrounding organs to be delineated in real-time. While CT allows for visualisation of the entire ice ball, ultrasound has a limitation with its inner margin, which is limited by acoustic shadowing. Similarly, post-ablation complications such as bleeding are better assessed on CT than ultrasound [28].

Post-ablation syndrome has been studied in more depth for RFA. In one prospective study [16], one-third of patients had the complete spectrum of post-ablation syndrome, with low-grade fever and flu-like symptoms (temperature > 37 °C). There appears to be a lower incidence of complete post-ablation syndrome following cryoablation with only six patients (9%) having a complete post-ablation syndrome.

However, the results for patients experiencing at least one of the components of post-ablation syndrome are similar for our cryoablation cohort compared to other published data on RFA, which found 56% experienced either fever or flu-like symptoms. In this study, 63% developed either flu-like symptoms (*n* = 39) or fever (*n* = 1). In a previous RFA study [16], a total of 42% of patients developed a low-grade fever (between 37 and 39 °C) compared to 11% in our cryoablation cohort. If the main driver of fever was from the inflammatory response secondary to the tissue death caused by the ablation one would expect a similar proportion of patients undergoing RFA and cryoablation to have fever, given the previous study [16] had a mean renal tumour size of 3.1 cm and this study had a mean tumour size of 3 cm. It is unclear as to what may result in this discrepancy in the incidence of post-cryoablation fever and it is unknown whether the cooling effect from the cryoprobes during the procedure reduces the effect from local inflammatory mediators caused by the cell death. The mean temperature in the cryoablation group with fever peaked on day 3 and self-limiting following this, in the same trend as the fever experienced following RFA [16].

Our data appears to suggest the presence of fever may exacerbate the severity of flu-like symptoms that are experienced with a higher mean grade of flu-like symptoms especially on day 1 and 3 for patients with concomitant fever. We acknowledge the data does not demonstrate causation rather an association. This correlation was not statistically significant on our t test; however, this is likely due to the small sample of patients we had, particularly in the fever group. Similarly, no significant statistical correlation was found between the total cryoablation time and the number of cryoprobes with flu-like symptoms or pain. Theoretically, it would be expected that a larger volume of ablated tissue would result in a greater inflammatory response and therefore more severe post-ablation symptomatology. The lack of statistical correlation may relate to the sample size with only 18 patients (28%) where more than 6 cryoprobes were used, of which six patients (9%) required more than 10 cryoprobes.

It is vital to distinguish the clinical course of post-ablation syndrome after cryoablation with post-procedural complications such as concurrent infection. Overall, post-cryoablation syndrome symptoms tend to be mild, with a low-grade fever, and self-limiting and resolve spontaneously with conservative management compared to concurrent infection which should be considered in the event of unresolving or delayed onset fever as seen in the patient from our cohort who developed a concurrent chest infection and cellulitis.

The mean pain experienced in the first 10 days following cryoablation is similar to published post-RFA results and the cooling effect of cryoablation does not appear to have a therapeutic effect on overall pain although overall scores remain low (mean score 2.1) with 72% having 90–100% pain relief by day 10 with simple analgesics and 26 patients (41%) having no pain by day 10, without the use of any analgesics. Quantification of pain and evaluation of interference with lifestyle through effect on general activities and work remains subjective. Our survey highlights a trend in the pain experienced following cryoablation that peaks on day 3, slightly later than the pain following RFA, which peaked on day 1. The overall mean scores are, however, comparable. There was also no statistically significant difference in the pain experienced with or without concurrent fever.

The mean scores for interference on general activities (mean score 1.8 out of 10) and work (mean score 2.0) which both peaked on day 3 in our cryoablation group were similar to the published RFA data, with fever having no apparent influence on the severity of lifestyle interference. In Wah et al. [16], the overall interference with general activities was 5.2 and interference with work was 5.4; however, this included both liver and renal RFA where the patients with liver lesions experienced greater lifestyle interference thereby skewing the mean results [16]. No significant difference is noted in the lifestyle interference following cryoablation from our data compared to post-RFA published data.

This study is limited by its small sample size and subjectivity from the patient’s own measurement of their temperature and using a telephone survey to assess clinical symptoms and lifestyle interference. It does form the basis for a larger formal study to review such findings.

## Conclusion

The full spectrum of post-ablation syndrome following cryoablation occurs in approximately 9% of patients; however, 61% of patients experience flu-like symptoms in the first 10 days, which are self-limiting.

Patients should be reassured that such symptoms are often self-limiting and further investigation is only required when there is persistent or late onset fever indicating possible concurrent infection.
